# Focal and Generalized Patterns of Cerebral Cortical Veins Due to Non-Convulsive Status Epilepticus or Prolonged Seizure Episode after Convulsive Status Epilepticus – A MRI Study Using Susceptibility Weighted Imaging

**DOI:** 10.1371/journal.pone.0160495

**Published:** 2016-08-03

**Authors:** Rajeev Kumar Verma, Eugenio Abela, Kaspar Schindler, Heinz Krestel, Elisabeth Springer, Adrian Huber, Christian Weisstanner, Martinus Hauf, Jan Gralla, Roland Wiest

**Affiliations:** 1 University Institute for Diagnostic and Interventional Neuroradiology, Inselspital, University of Bern, Bern, Switzerland; 2 Institute of Radiology and Neuroradiology, Tiefenau Hospital, Bern, Switzerland; 3 Department of Neurology, Inselspital, University of Bern, Bern, Switzerland; 4 University Institute for Diagnostic and Interventional Radiology, Inselspital, University of Bern, Bern, Switzerland; University of Modena and Reggio Emilia, ITALY

## Abstract

**Objective:**

The aim of this study was to investigate variant patterns of cortical venous oxygenation during status epilepticus (SE) using susceptibility-weighted imaging (SWI).

**Methods:**

We analyzed magnetic resonance imaging (MRI) scans of 26 patients with clinically witnessed prolonged seizures and/or EEG-confirmed SE. All MRI exams encompassed SWI, dynamic susceptibility contrast perfusion MRI (MRI-DSC) and diffusion-weighted imaging (DWI). We aimed to identify distinct patterns of SWI signal alterations that revealed regional or global increases of cerebral blood flow (CBF) and DWI restrictions. We hypothesized that SWI-related oxygenation patterns reflect ictal or postictal patterns that resemble SE or sequelae of seizures.

**Results:**

Sixteen patients were examined during nonconvulsive status epilepticus (NCSE) as confirmed by EEG, a further ten patients suffered from witnessed and prolonged seizure episode ahead of imaging without initial EEG. MRI patterns of 15 of the 26 patients revealed generalized hyperoxygenation by SWI in keeping with either global or multifocal cortical hyperperfusion. Eight patients revealed a focal hyperoxygenation pattern related to focal CBF increase and three patients showed a focal deoxygenation pattern related to focal CBF decrease.

**Conclusions:**

SWI-related hyper- and deoxygenation patterns resemble ictal and postictal CBF changes within a range from globally increased to focally decreased perfusion. In all 26 patients the SWI patterns were in keeping with ictal hyperperfusion (hyperoxygenation patterns) or postictal hypoperfusion (deoxygenation patterns) respectively. A new finding of this study is that cortical venous patterns in SWI can be not only focally, but globally attenuated. SWI may thus be considered as an alternative contrast-free MR sequence to identify perfusion changes related to ictal or postictal conditions.

## Introduction

“Time is brain” is a phrase commonly used to emphasize the fact that neurons are prone to immediate hypoxic cell death in acute stroke if tissue is deprived of oxygen and nutrients. Although neuronal death due to epilepsy is less frequently recognized than neuronal death resulting from acute stroke, it is a significant feature of epilepsy in humans. However, clear epidemiologic evidence of the deleterious effects of status epilepticus (SE) is still lacking. Imaging sequelae of seizures encompass widespread and frequently unspecific patterns including high and low T2-signal, leptomeningeal contrast-enhancement, hyper/-hypoperfusion or restricted diffusion [1]. Although routine MRI may frequently be normal when examined during non-convulsive SE (NCSE) or during prolonged seizure episode (PSE), it may later evolve into secondary brain atrophy. Neuronal loss has been confirmed in postmortem studies in atrophic brain areas after NCSE, suggesting that neuronal loss and cerebral atrophy can result from SE in humans [2]. Neuronal injury and death is not only related to excessive neuronal synchronous activity and subsequent breakdown of the neuronal energy supply, but also to the type of seizures (e.g. focal dyscognitive SE) and excitotoxic mechanisms in remote areas of the brain [3]. Since early diagnosis of NCSE and PSE and persisting ictal activity in the emergency room is necessary to prevent secondary neuronal injury, several imaging biomarkers, such as diffusion-weighted imaging (DWI) [4], perfusion MR [5,6] and computed tomography-based perfusion imaging [7] have been investigated in clinical practice, with a reported sensitivity of 78% during ictal and 37% during postictal conditions [8].

Diffusion restriction in ischemia and epilepsy appear to occur under very different cerebral blood flow (CBF) and metabolic conditions. In contrast to ischemic stroke, an increased metabolic demand during seizures is coupled to an increase in CBF, thus the cellular energy status is maintained. Ictal perfusion during epileptic seizures is reflected by increased metabolism and regional CBF along with synchronized firing of neurons comprising the symptomatogenic zone, rather than the seizure onset zone [9,10]. T2*-perfusion imaging requires an exogenous paramagnetic contrast agent (gadolinium), making it partially invasive and unsuitable for repeated measurements. Arterial spin labeling perfusion imaging, in contrast, is completely non-invasive and has been shown to enable the radiologist to detect ictal and early post-ictal perfusion changes in epilepsy patients [11]. However, perfusion changes are only indirectly coupled to epileptic discharges and are insensitive to variations in local energy consumption. Susceptibility-weighted imaging (SWI), which is a high-resolution 3D phase enhanced gradient echo method, offers added value as it identifies different tissue properties and states in both magnitude and phase images. A recent study demonstrated that focal hyperperfusion patterns during NCSE were associated with focal changes in the deoxy-Hb along pial veins in SWI, indicating increased metabolic demands owing to abnormal neuronal hypersynchronization even in patients with normal DWI [12].

The current study investigated patterns of global versus regional perfusion changes associated with oxy- versus deoxygenating (reflected by patterns of altered oxygenation of pial veins in SWI). We hypothesized that complex patterns of ictal metabolic changes in the brain may be identified by analyzing: (1) decreases in phase (SWI) reflected by widespread increases in oxygen saturation that indicate compensated hyperperfusion during SE or ictus, and (2) increases in phase that reflect decreases in oxygenation and indicate insufficient neuronal energy supply and either postictal conditions or transient neuronal exhaustion.

## Materials and Methods

### Patient data

The study was approved by the local ethics committee (cantonal ethics committee Bern, Switzerland). Because of the retrospective study design the informed consent was waived. The data analysis was performed after data encryption. We analyzed patients admitted to our institution by the emergency department between July 2010 and October 2013. Patients underwent MRI immediately after a neurological examination to identify acute neurological symptoms related to the seizure and their respective pathology. Inclusion criteria were: (1) a clinically witnessed general convulsive seizure with a prolonged seizure episode (PSE) in accordance with an operational definition of >5 minutes of continuous seizure activity or intermittent seizure activity, or (2) EEG-confirmed persisting NCSE, (3) an MRI with DWI, SWI and dynamic susceptibility contrast (DSC) MRI at emergency admission. Patients were excluded if image quality was poor, e.g. due to motion artifacts.

We denoted the subgroup with a witnessed convulsive seizure and subsequent continuous or intermittent ictal and/or postictal symptoms as “PSE” patient group based on the clinical observations of the neurologist in charge during the emergency setting.

### Data acquisition

Imaging studies were performed with 1.5T and 3T Siemens MRI (Magnetom Avanto and Magnetom Trio; Siemens Medical Solution, Erlangen, Germany) using a standard 12-channel head coil. The MRI protocol was part of our domestic emergency protocol, which includes the following sequences: axial DWI, axial T2 SE, fluid-attenuated inversion recovery (FLAIR), time of flight angiography, perfusion imaging, contrast-enhanced angiography of the cervical and intracranial arteries, and an axial T1 SE post-contrast. For the 1.5 T MRI the SWI parameters were: repetition time (TR) 49 ms, echo time (TE) 40 ms, number of averages 1, FoV read 230 mm, FoV phase 81.3%, voxel size 1.1×0.9×1.8 mm, flip angle 15°, acquisition time 2:59 min. Perfusion imaging parameters (DSC): TR 1410 ms, TE 30 ms, number of averages 1, FoV read 230 mm, FoV phase 100%, voxel size 1.8x1.8x5.0 mm, flip angle 90°, acquisition time 2:00 min. DWI parameters: TR 3000 ms, TE 89 ms, number of averages 4, FoV read 230ms, FoV phase 100%, voxel size 1.2x1.2x5.0 mm, acquisition time 1:35 min. For the 3 Tesla scanner the SWI parameters were as follows: TR 27 ms, TE 20 ms, number of averages 1, FoV read 230 mm, FoV phase 75.0%, voxel size 0.9×0.9×2.0 mm, flip angle 15°, acquisition time 2:59 min. A standard perfusion imaging sequence was used with the following parameters (DSCE): TR 1400 ms, TE 29 ms, number of averages 1, FoV read 230 mm, FoV phase 100%, voxel size 1.8x1.8x5.0 mm, flip angle 90°, acquisition time 1:59 min. DWI parameters: TR 3500 ms, TE 89 ms, number of averages 4, FoV read 230 mm, FoV phase 100%, voxel size 1.8x1.8x4.0 mm, acquisition time 1:15 min. The SWI and minimum intensity projection images were generated automatically by the scanner software.

### Data analysis

Two neuroradiologists with more than 10 years of experience (R.K.V. and R.W.) blinded to patient history, neurological symptoms or lateralizing signs, except for disclosure of an ictal or postictal condition, reviewed the imaging data. The images were evaluated on our picture archiving and communication system. All sequences were evaluated consecutively, starting with SWI, followed by CBF maps and DWI. For SWI analysis, global or focal attenuation or patency of the cortical veins (due to a higher oxyhemoglobin content) or intensification (due to a higher deoxyhemoglobin content) compared to a reference image were rated as either “global”, “multifocal” or “regional” increase or decrease. For semi-quantitative analysis of the perfusion patterns and quantification of CBF, the Olea PerfScape^®^ V2.0 was used (Olea Medical SA, France). Color-coded parametric maps of rCBF were obtained from the DSC data. According to the color-coding, parenchymal areas with an increase or decrease in rCBF maps were denoted, indicating a global or focal perfusion alteration reflecting altered metabolic demands. The areas of abnormal perfusion were marked using the OLEA grow region algorithm “magic wand” and the CBF values and their standard deviations were exported (in ml/100 g/min). The corresponding regions of the contralateral hemisphere were used as a reference. If visual analysis of CBF revealed no alterations, CBF analysis was referenced to corresponding regions in the frontal and superior temporal lobe on both sidesto perform a standardized analysis procedure, assuming that the temporal lobe is frequently affected during status epilepticus [4].

DWI images were visually analyzed to identify brain areas encompassing a cortical diffusion restriction. In cases of divergent results between the two raters, a consensus reading was established in a final reading session. If available, EEG results of patients were used as a reference and checked for overlap with the MRI-related regional abnormalities on a lobar level (Table 1). According to the imaging findings in the SWI and the color-coded maps of the perfusion images, patients were subdivided into four groups: 1. global hyperoxygenation in SWI and symmetrically normal perfusion (color-coded maps), 2. global hyperperfusion in SWI and multifocal hyperperfusion (color-coded maps), 3. focal hyperoxygenation in SWI and focal hyperperfusion, and 4. focal hypooxygenation and focal hypoperfusion.

**Table 1:** Detailed patient data. Detailed information on patients including underlying seizure pathology, findings in SWI, perfusion imaging and DWI. Division of patients according to the findings in SWI and perfusion imaging: patients 1–8, group 1; patients 9–15, group 2; patients 16–23, group 3; patients 24–26, group 4.

PatientNo.	Sex, age	Underlying seizure pathology	Cortical veins in SWI	CBF (color coded)	CBF±SD (ill- or normal perfusion appearance in color-coded maps)	CBF±SD (contralateral side with normal perfusion appearance in color-coded maps)	DWI DR	PSE/NCSE	EEG
1	F, 58	Encephalitis, no cerebral swelling	none	symmetric	93.26±22.79	110.78±31.65	none	PSE	Y
2	F, 78	non-lesional	rarified	symmetric	73.04±4.44	74.15±0.40	none	PSE	Y
3	M, 82	non-lesional	none	symmetric	85.66±49.38	62.10±46.45	none	NCSE	Y
4	M, 19	Encephalitis, no cerebral swelling	none	symmetric	96.64±46.76	113.03±49.44	none	PSE	N
5	F, 63	H/o infarction left	none	symmetric	146.59±40.05	151.71±35.60	none	PSE	N
6	M, 41	non-lesional	rarified	symmetric	102.37±16.86	100.8±30.04	none	NCSE	Y
7	F, 60	H/o several infarctions	none	symmetric	130.72±25.80	141.60±8.71	none	PSE	N
8	F, 63	cerebral lymphoma	none	symmetric	93.17±27.39	89.17±35.77	none	NCSE	Y
Mean CBF± values (group 1)					102.68±29.18	105.41±29.76	none		
9	F, 60	non-lesional	rarified	multifocally increased	82.73±28.55	50.28±25.32	none	NCSE	Y
10	F, 62	H/o aneurysm clipping and SAH	none	multifocally increased	138.82±16.88	63.26±5.24	none	NCSE	Y
11	F, 78	non-lesional	none	multifocally increased	110.31±15.14	63.36±0.78	focally	NCSE	Y
12	F, 84	parenchymal gliosis	none	multifocally increased	85.15±26.08	44.00±11.26	focally	NCSE	Y
13	M, 73	parenchymal gliosis after hemorrhage	none	multifocally increased	166.95±45.12	60.70±7.61	none	PSE	N
14	M, 57	glioblastoma multiforme	none	multifocally increased	108.10±29.92	48.96±22.21	focally	NCSE	Y
15	M, 72	metastasis of unknown primary tumor	rarified	multifocally increased	139.26±32.29	59.29±20.95	focally	NCSE	Y
Mean CBF± values (group 2)					118.76±27.71	55.69±13.33	none		
16	M, 55	parenchymal gliosis after traumatic hemorrhage	focally decreased	focally increased	94.78±25.85	35.72±7.60	Focally correlating with SWI	NCSE	Y
17	F, 22	non-lesional	focally decreased	focally increased	140.53±39.61	75.29±47.25	focally correlating with SWI	NCSE	Y
18	F, 67	non-lesional	focally decreased	focally increased	96.79±30.42	51.29±2.94	Focally correlating with SWI	NCSE	Y
19	F, 87	non-lesional	focally decreased	focally increased	87.49±23.68	37.20±9.57	Focally correlating with SWI	NCSE	Y
20	M, 4	Fever	focally decreased	focally increased	157.64±56.49	41.98±2.87	none	NCSE	Y
21	F, 73	metastasis of bronchial carcinoma	focally decreased	focally increased	135.02±41.16	51.55±14.36	none	NCSE	Y
22	F, 6	non-lesional	focally decreased	focally increased	178.19±51.18	97.41±43.55	none	NCSE	Y
23	F, 67	metastasis of unknown primary tumor	focally decreased	focally increased	96.18±24.60	47.78±15.82	none	PSE	N
Mean CBF± values (group 3)					123.33±36.60	54.78±18.00	none		
24	M, 77	non-lesional	focally increased	focally decreased	34.85±10.58	67.02±41.72	none	PSE	N
25	F, 65	hippocampal sclerosis	focally increased	focally decreased	25.62±12.06	58.68±24.75	none	PSE	N
26	M, 79	non-lesional	focally increased	focally decreased	19.81±13.17	40.07±14.53	none	PSE	N
Mean CBF± values (group 4)					26.76±11.94	55.26±27.00	none		

CBF, cerebral blood flow; PSE, prolonged seizure episode; DR, diffusion restriction; DWI, diffusion weighted imaging; EEG, electroencephalography; H/o, history of; NCSE, non-convulsive status epilepticus; SAH, subarachnoid hemorrhage; SD, standard deviation; SWI, susceptibility weighted imaging.

### Statistical analysis

Statistical analysis of CBF values was performed with IBM SPSS Version 20.

A within-group statistical analysis was performed on the semi-quantitative CBF maps to account for focal versus global perfusion changes. Within-group analysis encompassed the region-of-interest CBF measures of the hyper- or hypoperfused area (color-coded maps of groups 2–4) versus the normal-appearing corresponding region of the opposite hemisphere. In cases with symmetrical CBF patterns (group 1) the CBF values were referenced to the temporal lobe and compared between the hemispheres. Due to a given normal distribution a paired t-test was used to detect statistically significant differences between the hemispheres.

## Results

All clinical, imaging, and demographic details are summarized in Table 1. We identified 33 patients who underwent emergency MRI within 60 minutes after admission- or prior to EEG confirmation of the NCSE. Seven patients had to be excluded, 5 due to motion artifacts, 2 due to an incomplete study protocol. Twenty-six patients (15 female, 11 male; age range 4 to 87 years, mean 59.7 years) fulfilled the quality requirements for reading. NCSE was confirmed in all 16 patients by EEG after the MRI, 3/16 presenting with global CBF increase (1 with a structural epileptogenic lesion (SEL), 2 non-lesional (NL), 6/16 with multifocal CBF increase (4 with SEL, 2 NL) and 7/16 with focal CBF increase (2 SEL, 4 NL). All patients with a prolonged NCSE revealed a pattern of hyperoxygenation on SWI images. It is noteworthy that all focal CBF hyperperfusion patterns were reflected by focal hyperoxygenation on SWI (7/16), while both generalized and multifocally increased CBF patterns (9/16) were reflected by global hyperoxygenation patterns of SWI. The remaining 10 patients presented with PSE (2 with EEG after MRI, 8 without EEG) and varied considerably in their neuroimaging patterns. The two patients with PSE who received EEG revealing persisting ictal discharges after MRI presented with SWI and CBF patterns of global hyperoxygenation and hyperperfusion. Four patients with PSE not confirmed by EEG, presented with a similar pattern of global or multifocal hyperperfusion accompanied by global hyperoxygenation patterns on SWI. The remaining 4 patients presented either with focal hyperperfusion/hyperoxygenation or focal hypooxygenation/ hypoperfusion.

**Fig 1:**
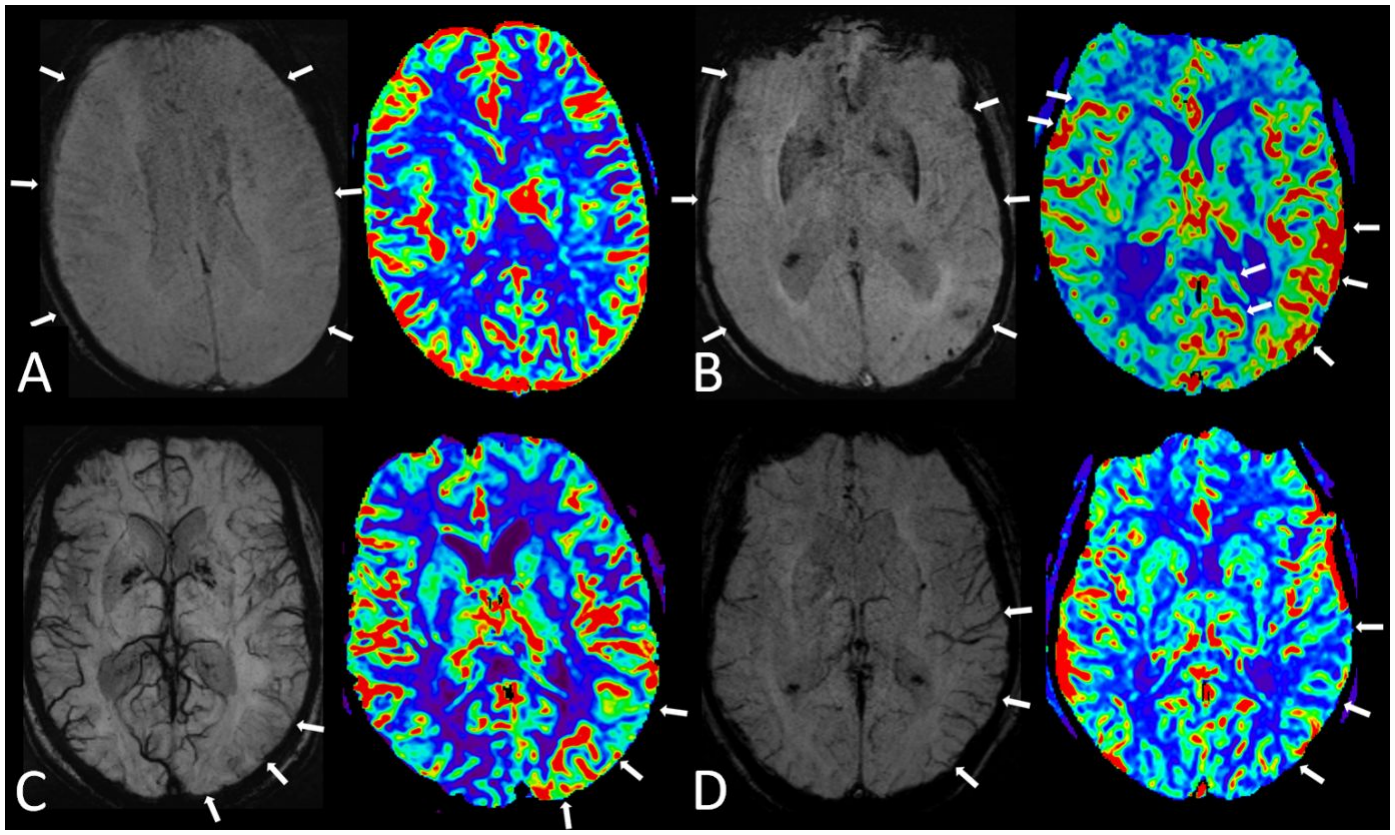
Division into subgroups according to the SWI and perfusion findings. (A) (group 1), in SWI the cortical veins are diminished (arrows) and the rCBF pattern in color-coded maps is symmetrical and reveals no abnormality (patient No. 5: Patient had a H/o left-sided watershed infarction. During MRI acquisition patient suffered from PSE after witnessed convulsive SE before MRI). (B) (group 2) again diminished cortical veins can be seen, but the rCBF pattern shows patchy multifocal hyperperfusion (patient No. 11: Patient had a NCSE during MRI with symptoms of acute aphasia, confusion and agitation). (C) (group 3) shows focally diminished veins with corresponding focal hyperperfusion (arrows); (patient No. 23: patient suffering from PSE during MRI with global aphasia and mild hemiparesis on the right). (D) (group 4) reveals left-sided prominent cortical veins with corresponding hypoperfusion (patient No. 24: Patient suffering from PSE with severe global aphasia during MRI).

For post-hoc analysis, we allocated each of the 26 patients according to their perfusion patterns to one of four distinct subgroups and analyzed their corresponding SWI patterns. The first group showed a symmetrical normal perfusion pattern (8/26 patients; Fig 1A), with rCBF upregulations in the temporal neocortex (mean rCBF = 102.68 (SD 24.2) on the right and 105.42 (SD 30.9) ml/100 g/min on the left; p = 0.578). On SWI the cortical veins appeared globally hyperoxygenated, with no diffusion restriction. These patterns were further referred as either “none” or “rarified” cortical veins on SWI. “None” indicated no visibility of any cortical veins on the SWI maximum intensity projections. “Rarified” indicated patterns where some cortical remnants of deoxygenation were present within a very large degree of diamagnetic patterns along cortical veins. Three patients presented with NCSE and five with a prolonged seizure.

The second group comprised 7 patients who presented with a multifocal perfusion increase (Fig 1B), 4 of them with focal diffusion restriction. Regional CBF increases (mean 118.76 ml/100 g/min (SD 31.0) indicated persistent ictal activity. The normal-appearing contralateral areas showed a mean CBF within normal ranges (55.69 ml/100 g/min; SD 7.8; p = 0.001). The corresponding SWI pattern indicated global hyperoxygenation.

In the third group, 8 subjects presented with focal CBF increase. Seven of the 8 patients had a prolonged NCSE and 1 had a prolonged seizure. Four of these 8 patients showed corresponding focal diffusion restriction. The SWI pattern resembled focal hyperoxygenation. CBF analysis revealed a focally associated CBF increase and normal CBF values in the corresponding contralateral cortex (average CBF 123.32 ml/100 g/min (SD 34.1) and 54.77 ml/100 g/min (SD 21.2), p<0.001); see Fig 1C).

**Fig 2:**
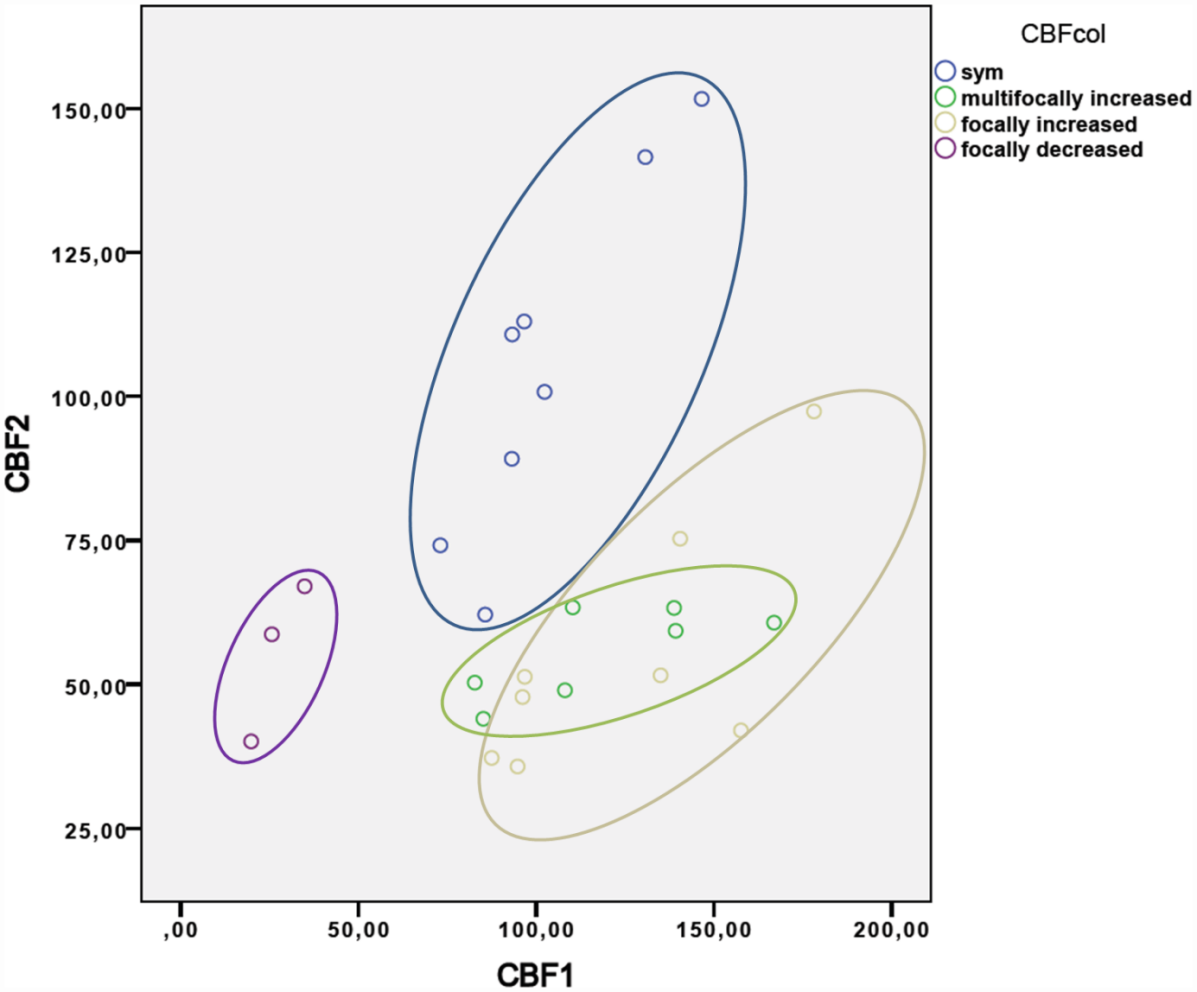
Scatter plot of quantified CBF. Each patient group is represented in an oval: group 1, blue oval; group 2, green oval; group 3, grey oval; group 4, purple oval. x-axis (CBF1): CBF value in ml/100 g/min of ill-perfused parenchyma y-axis (CBF2): CBF value in ml/100 g/min of the contralateral side. Group 1 shows a global hyperperfusion with mostly high CBF values in both hemispheres (x- and y-axis: >75 ml/100 g/min). Groups 2 and 3 show mainly unilateral higher CBF values (x-axis: >75 ml/100 g/min) and mostly normal CBF values on the opposite side (y-axis: around 50 ml/100 g/min). For group 4, CBF values are low in the ill-perfused area (x-axis: <50 ml/100 g/min), while the CBF values on the contralateral side are mostly in the normal range (y-axis: around 50 ml/100 g/min).

The fourth group comprising 3 patients presented with regional hypoperfusion with corresponding focal deoxygenation on SWI without diffusion restriction. Quantitative CBF analysis indicated regional hypoperfusion contrasted by normal values within the contralateral hemisphere (CBF = 26.76 ml/100 g/min (SD 7.6) and 55.26 ml/100 g/min (SD 13.8), respectively (p = 0.02); see Fig 1D). An overview of CBF patterns and numeric analysis, as well as a scatter plot encompassing quantified CBF values is provided in Table 2 and Fig 2, respectively.

**Table 2:** Overview of group findings. Division of patients into groups according to the findings in SWI and perfusion imaging.

Group	No. of patients	SWI pattern	rCBF pattern (color-coded maps)	Quantified CBF ml/100g/min (symptomatogenic)	Quantified CBF ml/100g/min (opposite)	p-value (laterality of CBF values)	Fig No.
1	8/26	Global hyperoxygenation	symmetric increase	102.7 (SD24.2)	105.4 (SD30.9)	0.578	1A
2	7/26	Global hyperoxygenation	multifocal increase	118.8 (SD31.0)	55.9 (SD7.8)	0.001*	1B
3	8/26	Focal hyperoxygenation	focal increase	123.3 (SD34.1)	54.8 (SD21.2)	<0.001*	1C
4	3/26	Focal hypooxygenation	focal decrease	26.8 (SD7.6)	55.3 (SD13.8)	0.02*	1D

Group 2 to 4 show significant differences in comparison of hemispheres, while in group 1 no significant difference is seen due to a global hyperperfusion in both hemispheres.

* Quantified CBF results with a statistically significant difference from the contralateral.

## Discussion

This retrospective study analyzed perfusion patterns associated with NCSE and PSE and their effect on venous oxygen extraction as detectable by SWI and their associated appearance of cortical veins. Considering the relatively high numbers of patients admitted to emergency rooms with altered mental state of unclear origin, a non-invasive imaging marker that identifies critical patterns of perfusion abnormalities associated with NCSE and PSE activity might support therapeutic decisions and better outcomes for patients. While PSE are frequently diagnosed based on their initial clinical observations, NCSE may be obscured at the time of admission to an emergency department. NCSE has an estimated incidence of 15/100,000 for all forms of SE [13]. NCSE accounts for 25% of all SE and for 9% of conditions that are associated with impairment of mental state and cognition at emergency admission. Furthermore, NCSE is present in 8% of patients with unclear comatose state without overt clinical signs of continuous epileptic activity, with an associated mortality of 18% [14].Focal NCSE is reported to be an independent predictor of high mortality, high morbidity and occurrence of refractory SE [15]. Generalized NCSE carries a low risk of mortality and is considered to be a more benign variant of NCSE [16,17]. The overall incidence of PSE in adults and children is estimated to be 17/100,000 with a mortality of up to 9.3% [18].

We identified four distinct patterns of altered oxygenation in patients with SE (DSCE-imaging and SWI): (1) widespread increased oxygenation following bihemispheric generalized cortical hyperperfusion and generally diminished cortical veins in SWI, (2) widespread increased oxygenation due to SE with multifocal hyperperfusion and again generally diminished cortical veins in SWI, (3) focally increased cortical oxygenation following focal cortical hyperperfusion and focally diminished veins in SWI, and (4) focally increased deoxygenation following focal cortico-subcortical hypoperfusion and focally amplified cortical veins in SWI. Although these patterns are presumably only “snapshots” of dynamic ictal and postictal changes and reflect only the metabolic state of the brain during the sequence acquisition, they are worth taking into consideration. While we observed a consistent upregulation of CBF associated with hyperoxygenated cortical SWI pattern in all patients with global increases of the metabolic demands during PSE or NCSE, DWI restrictions were focal and less consistent (4/15 patients). In patients with focal hyperoxygenation patterns associated with focal CBF increases, DWI also remained negative in 4/8 cases and must thus be considered as an unreliable marker of metabolic upregulation associated with NCSE and PSE. This finding may be explained by compensated metabolic demands due to the upregulated perfusion and less severe susceptibility of neocortical brain regions and duration of the event, where ictal hyperperfusion is confined to continuous seizure activity and tends to normalize after the cessation of the ictal activity [19,20]. Because hemodynamic correlates of epileptic seizures reflect a response to excessive neuronal activity, the perfusion patterns resemble the areas of the symptomatogenic zone during the spread of an epileptic seizure [21–23], whereas DWI restriction must be considered as markers of consecutive hypoxic damage [24].

Perfusion imaging is increasingly recognized as an imaging biomarker for focal epileptic activity or postictal dysfunction. However, it may be of limited value if generalized SE or focal alternating patterns of increased neuronal activity and depression elude visual perfusion analysis in a clinical setting [25]. Consistent with EEG changes, CBF patterns may differ even while patients remain clinically symptomatic (i.e. showing either focal neurological deficits or impaired consciousness) and also during a range of “boundary conditions” where there is ictal activity without obvious clinical seizures [7]. Following previous studies on perfusion imaging [26,27], we hypothesize that diamagnetic SWI patterns reflect neuronal hyperexcitation in case of persistent epileptic activity, whereas postictal hypoactivity may be reflected by local paramagnetic effects on SWI. Furthermore, cortical swelling may be associated with astroglio-vascular responses to status epilepticus and early vasogenic edema. The latter may be associated with increase in net brain water content, leading to a rise in intracerebral pressure and extracellular space expansion leading to attenuated venous appearance.

**Fig 3:**
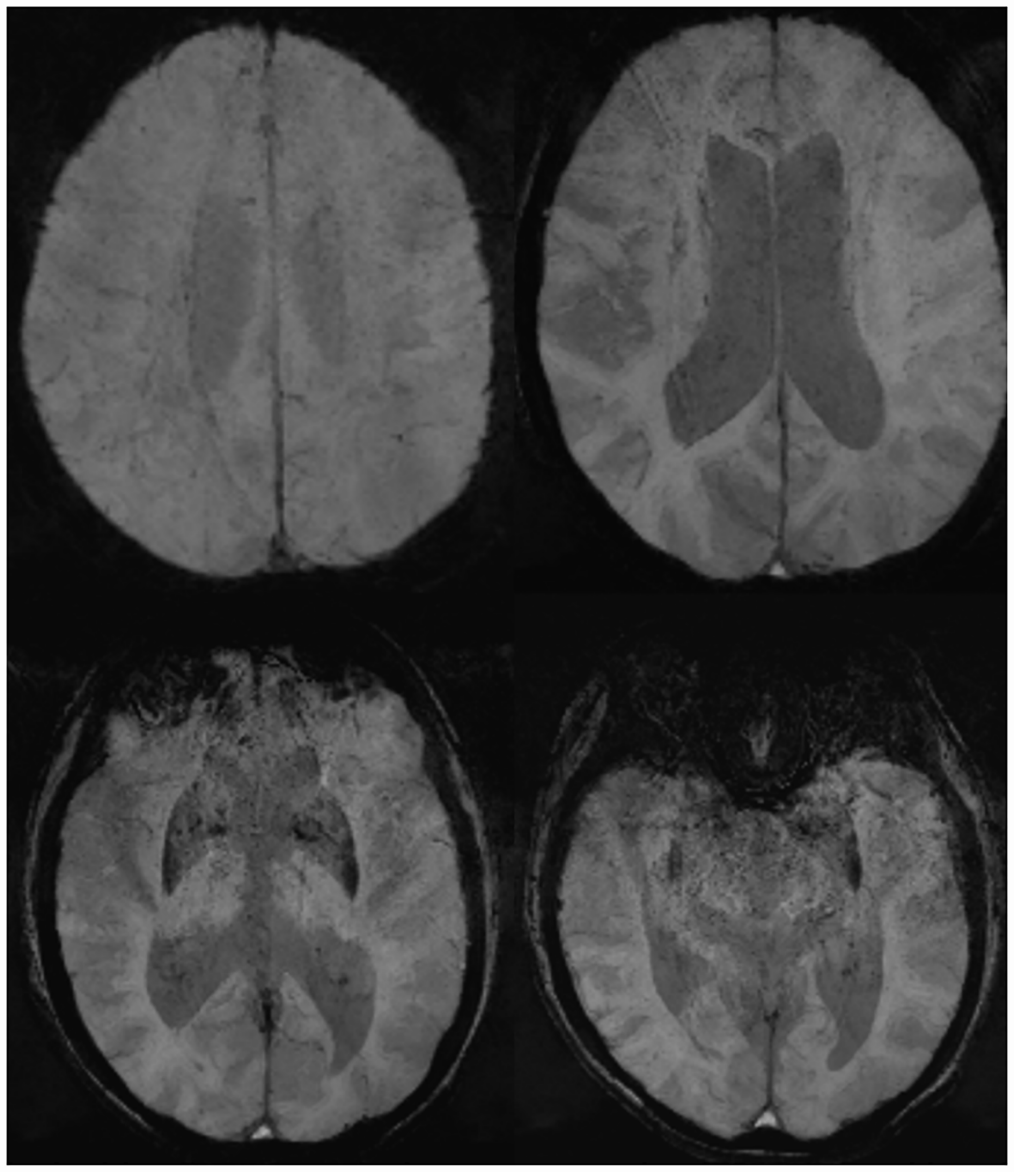
SWI Serial images of a patient with PSE. Globally diminished corticals veins due to global hyperperfusion in patients with NCSE or PSE is a new finding of this study. This is an example of a 78 y old female with NCSE (patient no. 11). Cortical veins are not visible in the minimum intensity projection SW images.

SWI can be used to qualitatively estimate the diamagnetic versus paramagnetic properties and thus to indirectly estimate the oxygenation level and extraction fraction in SE during postictal conditions or during focal ictal hyperactivity [12]. The novelty of our study is the observation of globally diminished cortical veins in SWI in 15 patients with NCSE/PSE), demonstrated by multifocal (7 patients) or global (8 patients) CBF increase in the color-coded maps (Fig 3). Here, SWI may act as an alternative non-invasive MR marker of increased neuronal activity during seizures. The qualitative visual inspection of rCBF maps that is frequently performed in daily practice may fail to interpret symmetric rCBF patterns appropriately in case of global hyperperfusion. Since DWI restriction was present in the minority of our patients, SWI might be the only sequence that would result in overt pathological findings.

### Study limitations

This study has several limitations. While the diagnosis of NCSE was supported by EEG in all cases, PSE diagnosis was based on the presence of a witnessed seizure. However, given the limited availability of EEG outside normal working hours and the low sensitivity of EEG during interictal recordings, this reflects current clinical practice and emphasizes the need for alternative non-invasive diagnostic tools that can be applied during the evaluation of SWI. We decided to waive a side-by-side correlation analysis of the EEG findings and perfusion patterns beyond the yes/no decision as to whether epileptic discharges were present. Further, we identified the time from arrival at our hospital to the MRI being less than 60 min, but the exact time of seizure onset before admission was not available in many cases. For correlation analyses, simultaneous EEG/fMRI or the recently proposed direct imaging of nonhemodynamic field effects using phase-cycled stimulus-induced rotary saturation (pc-SIRS) may be used in future studies [28]. Differential diagnosis of altered mental state is complex and includes various etiologies such as trauma, tumor, vascular disease, infections, metabolic disorders, and toxic encephalopathies. In a subset of patients these conditions may coincide with epileptic activity with hyperperfusion, thus fulfilling the diagnostic criteria for NCSE or PSE, e.g.we can not exclude, that the encephalitis in two patients caused the global cerebral hyperperfusion (Pt. No. 1 and No.4 in Table 1).

All patients presented with either negative symptoms as e.g. aphasia or focal motor symptoms, impaired vigilance or stupor and fluctuations of their symptomatology. Due to the retrospective character of the study, a more detailed description of the fluctuations of the symptomatology was not available.

In conclusion, this study investigated patterns of oxygenation using SWI in patients with NCSE and PSE. A new finding of this study is that cortical venous patterns in SWI can be either focally or globally attenuated in this cohort. SWI may thus be used as a complementary sequence to CBF maps and DWI to identify patients with altered mental state due to persisting ictal activity.

## Abbreviations

CBF: cerebral blood flow
DR: diffusion restriction
DSC: dynamic susceptibility contrast
DWI: diffusion-weighted imaging
EEG: electroencephalogram
NCSE: nonconvulsive status epilepticus
PSE: prolonged seizure episode
rCBF: regional cerebral blood flow
SE: status epilepticus
SWI: susceptibility-weighted imaging
